# Crack Width Evaluation of Cracked Mortar Specimen Using Gas Diffusion Characteristics

**DOI:** 10.3390/ma16020586

**Published:** 2023-01-06

**Authors:** Do-Keun Lee, Kyung-Joon Shin, Kwang-Myong Lee

**Affiliations:** 1 Civil Engineering, Chungnam National University, Daejeon 34134, Republic of Korea; 2 Department of Civil and Environmental Engineering, Sungkyunkwan University, Suwon 16419, Republic of Korea

**Keywords:** gas diffusion, crack, concrete, performance evaluation, self-healing

## Abstract

Several methods have been proposed currently for evaluating the crack width of a mortar specimen. Among these, the water permeability test is widely used to estimate crack width because water permeability is directly related to the average crack width of a specimen through which water passes. However, the viscosity of water makes precise crack width measurement challenging. The possible inflow (outflow) of foreign (healing) substances could affect the test results. To circumvent this limitation, this study proposes a gas diffusion test using oxygen rather than water as the medium. The proposed method includes a process that could compensate for gas diffusion from specimen parts other than the crack, allowing for a more precise estimation of crack width. The crack width can indeed be estimated with an error of 4% or less.

## 1. Introduction

As infrastructure across the globe ages, maintenance and repair costs for each country’s facilities and structures have steadily increased [1,2]. In general, the serviceability and durability of concrete structures deteriorate with age, and their maintenance costs increase as they age.

A crack is one of the most common phenomena of durability degradation for concrete structures. Since the amount of permeation of harmful substances is dependent on the width of the crack, the crack itself may pose a problem, and the crack width is a crucial factor in determining the durability of concrete structures. 

A water permeability test is commonly used to evaluate the crack width of a cracked specimen [3,4,5]. However, the viscosity of water makes precise crack width measurement difficult. In particular, when the crack width is small, obtaining reasonable results is challenging because water hardly passes through it [6]. 

Technologies, such as self-healing technology, which can increase the durability of concrete structures without requiring manual maintenance, garnered significant attention recently. Most studies on self-healing technology have focused on developing self-healing materials and technologies and enhancing the self-healing capability of materials [7].

As the study of self-healing advances, there is a growing need for more precise methods of evaluating the self-healing performance of materials. Microstructural analysis, water penetration tests, and ion transportation tests, which have been widely applied to typical concrete, are now being used by the industry to evaluate the performance of self-healing concrete [8]. However, there is a lack of standardized methods for assessing the performance of self-healing concrete, making it difficult to directly compare the performance evaluation results of different research teams because the tests were conducted differently [9].

Microstructural analysis based on microscopy and water penetration tests is currently one of the most widely used methods for evaluating self-healing performance [10]. Even within a single specimen, the width of concrete cracks can vary based on the observed area. Consequently, microscopy-based microstructural analysis results may vary depending on which portion of a specimen is examined. Therefore, visual inspection-based methods have limitations when analyzing the overall self-healing performance of concrete, especially for the entire specimen [9].

In contrast, water penetration tests can estimate the average self-healing rate of an entire concrete specimen; hence, this technique is now widely used to assess the crack width and self-healing performance of self-healing concrete. However, this method may result in reaction products escaping from cracks or foreign substances penetrating cracks during testing. The effect of the viscosity of the fluid used in the measurement must also be considered for accurate evaluation [6,10]. 

An alternative method using gas as a medium was proposed to overcome the limitations of water penetration tests when evaluating crack widths [8]. Based on a gas diffusion phenomenon through a crack, a crack’s width could be successfully evaluated using the proposed test method. Gas diffusion tests conducted on highly saturated specimens ruled out the possibility of gas diffusion through specimen components other than the crack. Therefore, only the amount of gas diffusing through the crack was considered in the evaluation. However, subsequent research revealed that the amount of gas diffusing through the cement-based matrix affected the determination of the crack diffusion coefficient of concrete, particularly for specimens with a low saturation level (i.e., high air permeability). This was found to result in estimation errors for crack width. Therefore, a more precise estimation of crack width for concrete specimens using gas diffusion experiments is required, considering both the gas diffusion behavior through a crack and that through the cement-based matrix. 

This study proposes an enhanced gas diffusion test method for estimating the width of a crack that could minimize the effect of gas transported from specimen parts other than the crack during gas diffusion experiments. In addition, mortar disk specimens were made to validate the proposed test method. Using the proposed gas diffusion test method, this study compared the estimated crack width with the experimentally determined crack width.

## 2. Overview of Gas Diffusion Experiment on a Concrete Specimen

### 2.1. Background to the Proposed Gas Diffusion Test Method

A cement-based composite, such as cement paste or mortar, possesses capillary pores that permit gas diffusion [11,12,13,14]. Numerous studies have examined the characteristics of these pores found in cement-based materials. For example, attempts have been constantly made to characterize such pores by evaluating the gas diffusion characteristics of non-cracked concrete [12]. According to Houaria [11], who studied gas diffusion in non-cracked concrete specimens, gas diffusion rates in cement-based materials are significantly influenced by the specimen’s saturation level. When the degree of saturation ranged from 55% to 100%, moisture within the specimen’s internal pores blocked the flow of gas, resulting in a significant decrease in the diffusion coefficient. Conversely, when the degree of saturation is low, the specimen’s gas diffusion coefficient increases significantly.

Lee et al. [9] proposed an experimental method for determining the width of a crack by measuring the amount of gas diffusing through a specimen’s crack. In the proposed method, gas diffusion through the matrix was disregarded because it occurred predominantly through the crack and not the matrix. Unlike previous studies in which both pressure and concentration gradients were applied [12], Lee’s study only applied diffusion by concentration gradients.

However, as the experimental results accumulated [15,16], it became evident that diffusion through the cement matrix also significantly affected the results, depending on the specimen’s condition. Specifically, when tests were conducted on specimens with a high degree of saturation, results were found to be relatively accurate; however, errors increased when tests were conducted on late-aged specimens with a low degree of saturation. Thus, these studies highlighted the need to measure the amount of gas passing only through a crack alone when attempting to estimate the crack width in mortar specimens containing a crack using gas diffusion experiments.

To achieve this, a method for increasing the degree of saturation of test specimens with cracks could be considered to prevent the diffusion of oxygen gas from the cement matrix. The greater the degree of saturation, the greater the effectiveness with which gas diffusion through the cement matrix can be blocked, thereby enhancing the accuracy of crack width estimation. However, the degree of saturation varies for each test specimen based on the curing conditions used. In addition, controlling the saturation level frequently requires a considerable amount of time. Thus, this method has been deemed unsuitable or less applicable. Therefore, this study proposes an enhanced experimental method for accurate crack width estimation regardless of the degree of saturation in a given specimen.

### 2.2. Relational Expressions for Crack Width Estimation

An index is required that reflects gas diffusion behavior, which varies in terms of crack width, to estimate the crack width based on diffusion phenomena within the specimen. The amount of gas diffusion per unit area remains constant regardless of the geometric shape of the diffusion cross-section. A relational expression correlating the crack width with the diffusion coefficient is necessary to estimate the crack width based on the diffusion coefficient. This study established this relational expression based on the ideal gas equation and Fick’s laws, as described by Lee et al. [9], who proposed the use of gas diffusion experiments for crack width estimation.

(1) $$K_{c}=\frac{V_{v}d}{L_{c}t}\cdot ln\left(\frac{C_{0}}{C}\right)$$

Here, $K_{c}$ is the crack diffusion coefficient of the specimen, $V_{v}$ is the volume of the test equipment, d is the crack depth, $L_{c}$ is the crack length, and t is the unit test time. Moreover, $C_{0}$ and C refer to the concentration difference between the inside and outside of the test equipment at the beginning and end of the test, respectively. The crack depth could be assumed to be a specimen thickness if actual measurements are not available.

Given the purpose of this crack width estimation, Equation (1) can be expressed as a function of the crack width:

(2) $$w=\alpha K_{c}$$

where α is the proportional coefficient of $K_{c}$ with respect to w. The α value may vary depending on the test conditions; however, this study used a value of 4.926 s/cm^2^ [15,17,18]. 

### 2.3. Diffusion through the Pores of the Cementitious Material

Cementitious materials are porous, and the mortar used in this study is a permeable material with both general and capillary pores. There have been reports of diffusion through the pores of cementitious materials, and the rate of diffusion is known to be related to the degree of saturation inside the medium [19]. During a gas diffusion test performed on a mortar specimen with a crack, oxygen from outside the specimen diffuses into the test device via the crack. In contrast, oxygen from outside or within the specimen pores simultaneously diffuses along the capillary pores. Therefore, to accurately estimate the crack width, we must exclude the portion of oxygen that diffuses through the matrix of the specimen and instead measure the change in oxygen concentration that only diffuses through the crack. 

**Figure 1:**
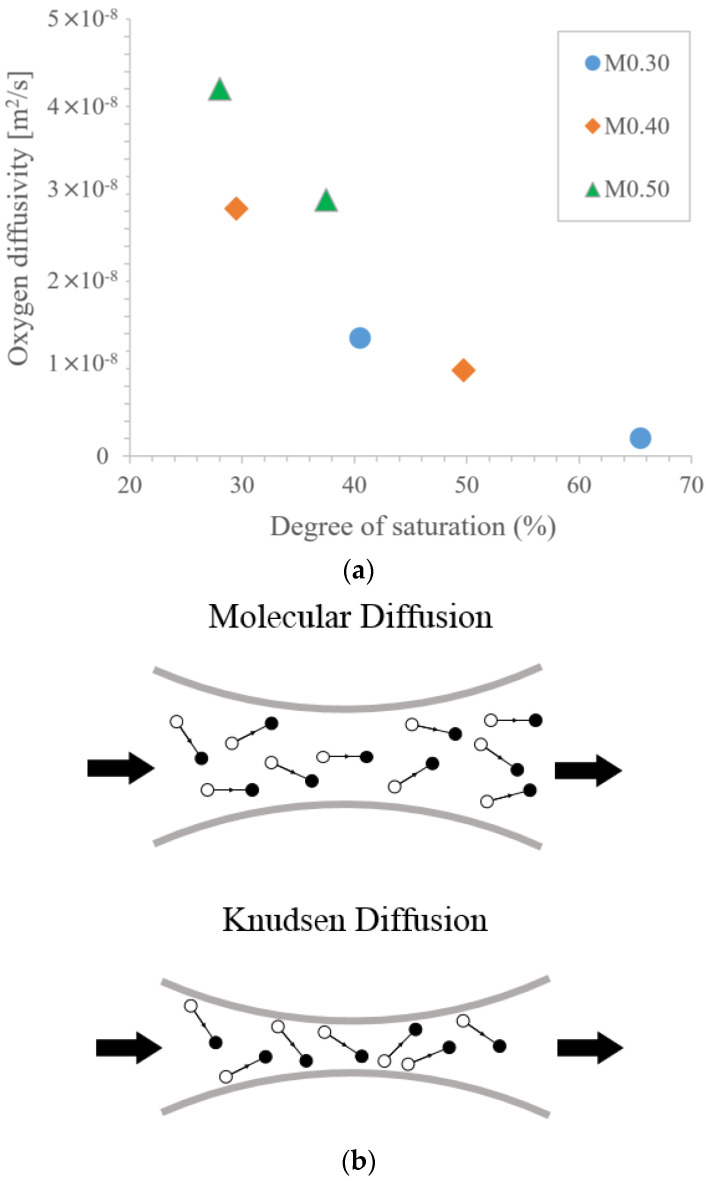
Gas diffusion properties, Chiara Villani et al., retouching [17]: (**a**) oxygen diffusivity as a function of the degree of saturation and (**b**) gas transport mechanism.

However, diffusion through the medium is divided into general diffusion and Knudsen diffusion, owing to the size of the pores, as shown in Figure 1 [16]. At high relative humidity (RH; greater than 80% RH), gel pores are filled with fluid; however, as relative humidity decreases, gel pores become empty and the connectivity between pores increases [19]. In pores smaller than 50 nm, Knudsen diffusion occurs due to collisions between molecules and the pore walls, not collisions between molecules. When the relative humidity inside the pores is decreased further (below 50% RH), the pore size and the diffusion area due to the collision of molecules increase.

Because a detailed diffusion mechanism through the medium varies concerning the saturation of pores and is affected by an excessive number of variables, mathematically modeling the diffusion of cement matrix considering the degree of saturation may be challenging. Thus, test procedures are proposed to compensate for diffusion through the matrix during the experiment. In addition, the surface of the specimen exposed to the external gas is blocked to simplify the cracked specimen’s diffusion boundary condition.

### 2.4. Measuring Diffusion Only through a Crack

The proposed method incorporates a procedure that can compensate for gas diffusion from specimen regions other than the crack. The proposed method consists of the following processes: (1) measuring the diffusion coefficient through the medium after conducting a diffusion test with a blocked crack (Phase 1); (2) measuring the diffusion coefficient simultaneously passing through the crack and the medium after opening the crack (Phase 2); and (3) measuring the crack diffusion coefficient through the crack by calculating the difference between these two diffusion coefficients. 

**Figure 2:**
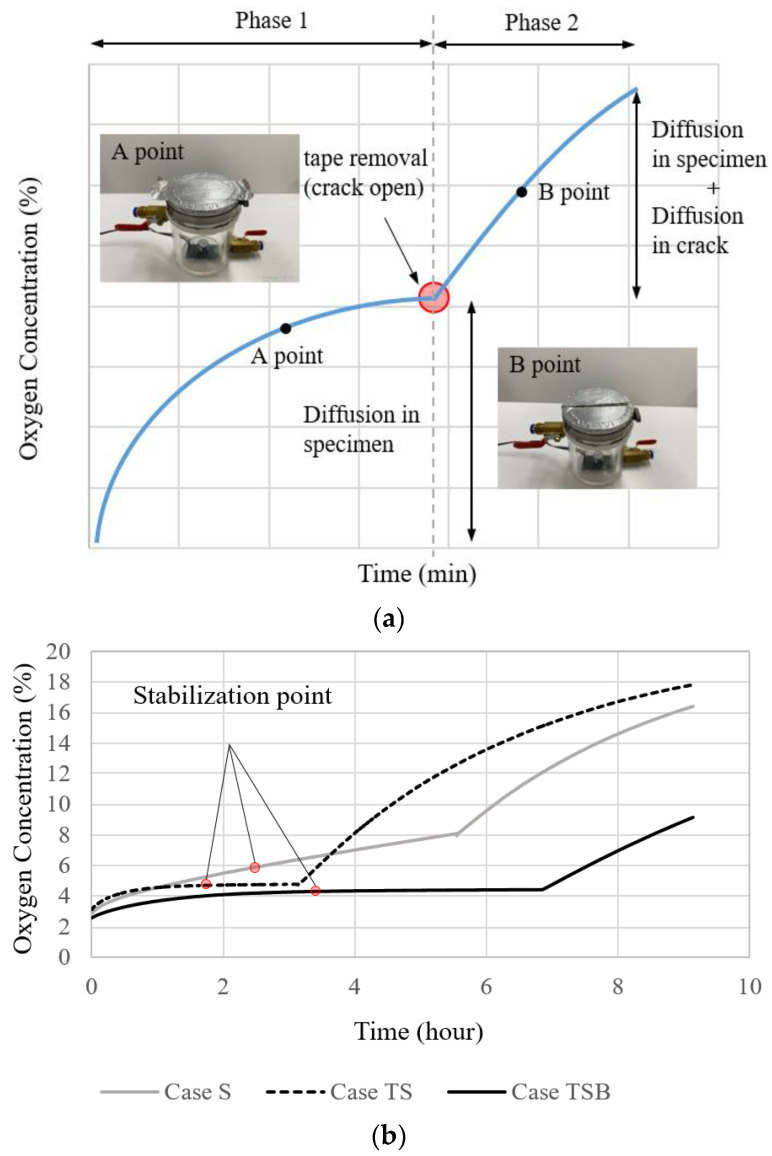
Concentration change in the experimental apparatus [18]: (**a**) overview of concentration changes in the test device with a crack closed or open, and (**b**) time to reach steady state in relation to the boundary condition of the specimen without pre-stabilization process.

The relationship between a gas diffusion coefficient and a crack width was investigated in a previous study [20], as depicted in Figure 2. The experiment demonstrates the change in concentration of the test device, in which the crack on the upper portion of the specimen was sealed with non-permeable aluminum tape at the start of the experiment and then removed after a specified period. Reportedly, the estimated crack widths from the gas diffusion test correlated well with the actually measured crack widths.

### 2.5. Pre-Stabilization to Shorten the Duration of the Gas Diffusion Test

As shown in Figure 2b, diffusion occurred nonlinearly at the beginning of the experiment until it reached a steady state. A certain amount of time was required from the start of the experiment until the diffusion reached a steady state and the concentration change became minimal. In Case TS, where the thickness of the specimen was 25 mm and both the upper and lower surfaces were coated, the stabilization of the slope of the graph took approximately 2 h. It is anticipated that 50 mm-thick specimens will require more time.

As the time required for the gas diffusion experiment increases, the inefficiency of the experiment increases as the number of specimens that can be examined in a given amount of time decreases. In addition, predicting how long the experiment will take is difficult because the time required to reach a steady state may vary depending on the specimen’s condition. 

Therefore, this study introduces a pre-stabilization method that can shorten the Phase 1 period, during which the concentration change in the test device reaches a steady state due to diffusion in the specimen. If the oxygen concentration of the specimen is adjusted in advance to a level comparable to the initial oxygen concentration in the test device, the specimen’s boundary condition is maintained so that the time required to reach a steady state is shortened. Obviously, the 24 h pre-stabilization process cannot maintain a constant concentration across the entire cross-section. However, the pretreatment could uniformly reduce the concentration in the specimen affected by the primary diffusion test.

## 3. Experimental Program

### 3.1. Outline of Test Procedures

**Figure 3:**
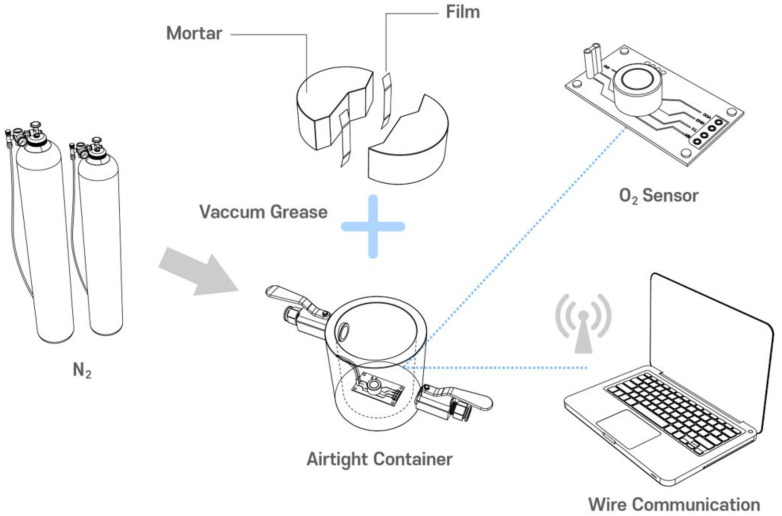
Overview of the experimental configuration and apparatus.

The examination comprises three steps. The first is the specimen preparation process. The second step is the pre-stabilization procedure, during which the prepared specimens are stored in low-concentration oxygen. The last step is the primary diffusion test, which induces oxygen diffusion through a crack by applying a concentration gradient to both sides of the prepared specimen. Figure 3 briefly describes the gas diffusion experiment procedure for a concrete specimen with a crack. 

### 3.2. Preparation of the Specimens

A specimen with a specified crack width can be created in multiple ways [21,22,23]. Basically, a splitting tensile test is utilized. However, the method could be subdivided into a method of restraining the specimen’s circumference so that it does not split after cracking and completely splitting the specimen and then clamping it again. This study employs the latter method, which is advantageous for producing specimens with identical conditions.

Several steps were required to produce the cracked specimens [6]. First, the mortar cylinders (Ø100 mm × 200 mm) were prepared. Next, these cylinders were demolded after 24 h and cured in a water bath at 20 °C until they reached the age at which cracking begins, which is 28 days. Once the cracking age was attained, the cylinders were sliced into a disc shape (Ø100 mm × 50 mm) and then split into two semicircular sections using a splitting tensile load. As shown in Figure 3, a flexible silicone rubber sheet was then attached to both ends of the cracked sections to induce a crack of a specified width. The desired crack widths were achieved using silicone rubber sheets of differing thicknesses. Finally, stainless steel bands were used to bind the split specimens together to maintain the desired crack widths.

**Figure 4:**
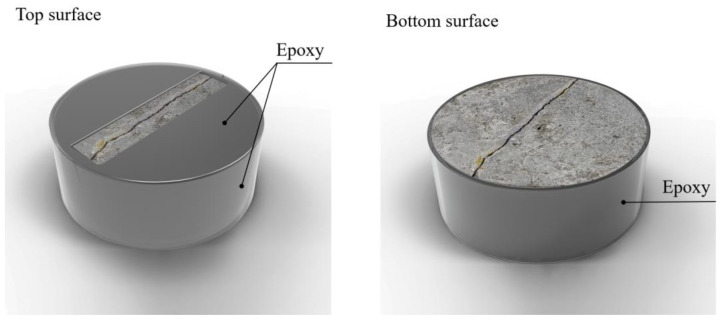
Epoxy coating of specimen.

Further, as shown in Figure 4, an epoxy material was applied to the specimen’s top and side surfaces. The coated specimen was exposed to air for approximately 24 h to cure the epoxy completely.

**Figure 5:**
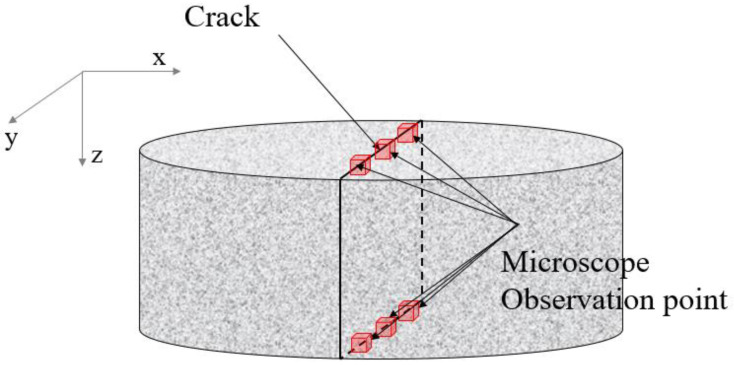
Crack width measurement using a microscope.

Each crack was then analyzed using a digital optical microscope. As depicted in Figure 5, the crack width was measured at three evenly spaced points along the crack line on the top and bottom surfaces of the specimens. The average crack width (*w_m_*) was determined using the arithmetic mean of the six crack width measurements. The crack data of the specimens are listed in Table 1. The appropriateness of the proposed method was demonstrated by comparing the measured crack widths to those estimated by the diffusion test.

### 3.3. Diffusion Test Method

**Figure 6:**
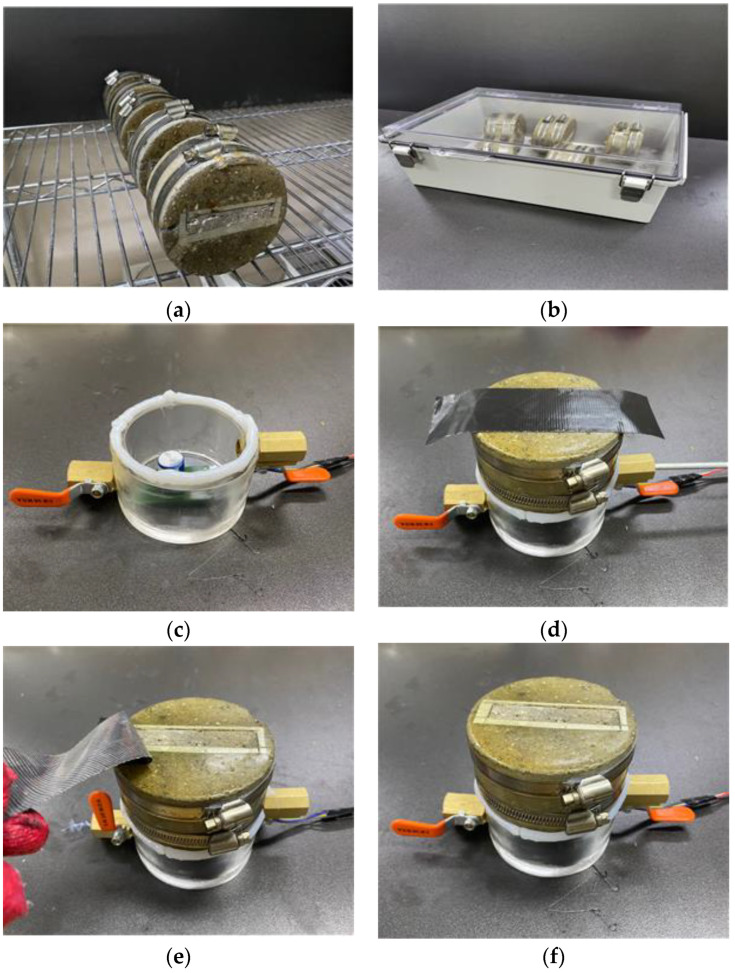
Experimental processes: (**a**) specimen creation; (**b**) pre-stabilization; (**c**) vacuum grease application; (**d**) specimen mounting and nitrogen injection; (**e**) tape removal during measurement; and (**f**) measurement following tape removal.

The pre-stabilization method reduces the oxygen concentration in the specimen to the initial level for the primary diffusion test by placing the specimen in a nitrogen-saturated container for 24 h. As depicted in Figure 6b, the specimen was placed in an airtight pretreatment container for pre-stabilization, and the initial oxygen concentration level inside the container was set to less than 3%. To monitor the pretreatment process, this study measured the change in oxygen concentration inside the pretreatment container. Subsequently, the pretreatment was terminated upon confirmation of an equilibrium state with no concentration change.

Following the pre-stabilization process, a gas diffusion test was conducted. The testing equipment consisted of a 250 mL acrylic cylinder container with a 40 mm inner diameter and a 50 mm height. The container was equipped with an inlet and outlet for nitrogen injection. The specimen’s crack was temporarily sealed with aluminum tape before attaching it with vacuum grease to the gas diffusion testing apparatus.

As nitrogen was injected into the test apparatus, an oxygen concentration gradient was generated between the inside and outside of the apparatus or between the inside of the apparatus and the internal pores of the specimen, causing oxygen to diffuse into the interior of the apparatus. Initially, the oxygen concentration within the device increased nonlinearly, but as the test progressed, it began to change linearly. This indicated that non-steady state diffusion occurred during the initial stages; however, the diffusion mode gradually transitioned into a steady state over time.

During the process, an oxygen detection sensor (SST Sensing Ltd., LuminOx model, Coatbridge, UK) with a 0.01% resolution installed inside the container measured variations in the oxygen concentration in real time. The outside oxygen concentration was determined by averaging the levels measured at the beginning and end of each test. After nitrogen was injected into the test device, data were collected every 1 s, and the aluminum tape was removed once the concentration change in the test device reached a steady state. The time required to reach a steady state was determined when the coefficient of determination for the linear equation exceeded 0.98 for 5 min.

### 3.4. Diffusion Coefficient Calculation for Crack Width Estimation

**Figure 7:**
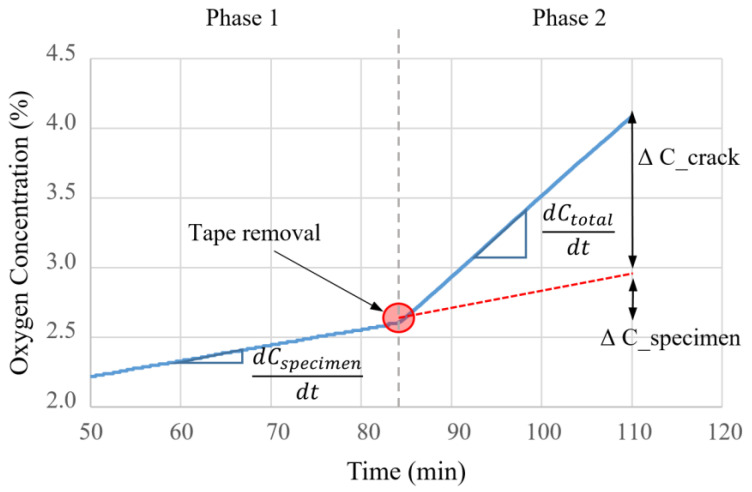
Schematic of variations in oxygen concentration before and after the start of diffusion through the crack.

Figure 7 depicts the amount of oxygen that changed in the test device for approximately 10 min prior to and after the removal of the tape.

When the tape is removed after the steady state has been reached and the crack is opened, additional diffusion from the outside atmosphere to the inside of the test device occurs through the crack, and the concentration gradient increases rapidly. Therefore, the diffusion coefficients were measured separately for the Phase 1 section, which reflects the matrix’s influence, and the Phase 2 section, which considers both the crack and the mortar matrix. Their difference was utilized to determine the crack diffusion coefficient when oxygen gas spreads solely through the crack. As shown in Equation (3), the gradient of the internal and external oxygen concentration difference and time were computed when the tape was attached (crack closed) and removed (crack open). By calculating the difference, we determined the log concentration-time gradient for oxygen gas diffusing from the exterior through the crack. Moreover, the crack diffusion coefficient was determined by substituting the log concentration-time gradient, *G*, into Equation (1).

(3) $$G={\left|\frac{\Delta \ln\left(c\right)}{\Delta t}\right|}_{open}-{\left|\frac{\Delta \ln\left(c\right)}{\Delta t}\right|}_{close}$$

where *G* is the log concentration-time gradient [s^−1^] for oxygen diffused through the crack, ${\left|\frac{\Delta \ln\left(c\right)}{\Delta t}\right|}_{open}$ and ${\left|\frac{\Delta \ln\left(c\right)}{\Delta t}\right|}_{close}$ refer to the log concentration-time gradients for the measurements before and after tape removal, respectively.

## 4. Results and Discussion

### 4.1. Estimation of Crack Width

**Figure 8:**
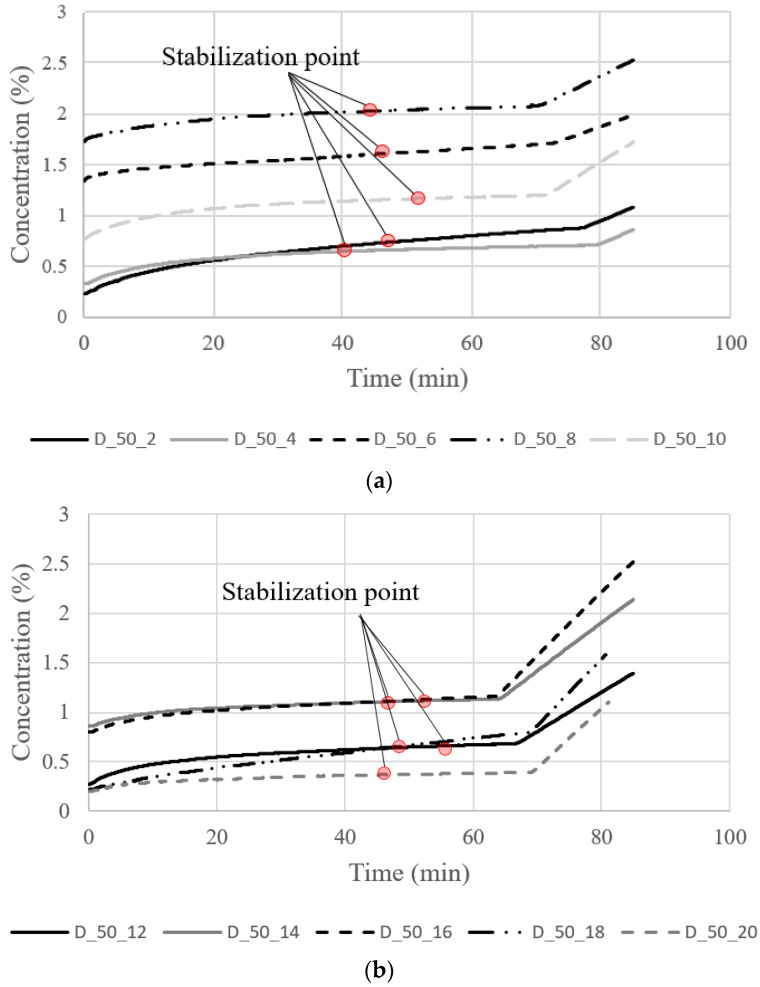
Concentration change in experimental equipment: (**a**) D_50_2–10; (**b**) D_50_12–20.

Figure 8 shows a graph showing the change in concentration in the test container during the gas diffusion test of even-numbered specimens from D_50_01 to D_50_20 with a thickness of 50 mm. There is no significant difference in the trend of results for D_25 series.

Despite pre-stabilization, a relatively rapid change is considered to occur in the observation period within 20 min because of the difference between the final concentration of oxygen in the specimen and the initial concentration of the diffusion test. However, the measured oxygen concentration converged linearly within 1 h for all samples. The coefficient of determination is at least 0.98, which indicates that the diffusion between the test device and the test specimen has reached a steady state.

The crack diffusion coefficients were calculated using Equations (1) and (3) based on the measured concentration data. Following, the crack widths for specimens D_25 and D_50 were estimated using Equation (2) with the measured crack diffusion coefficients. Here, the crack depth was assumed to be the specimen height. The crack length was the average value of the actual crack length measured from the top and bottom surface of the spcimen. Table 2 displays the difference between the actual crack widths measured by microscopic operation and the estimated widths.

The average errors of crack width estimated using the gas diffusion test were 2.89% and 4.02% for D_25 and D_50 specimens, respectively. Meanwhile, the maximum errors were 3.61% and 9.21% for D_25 and D_50 specimens, respectively. Five specimens in D_50 series were observed to have an error rate exceeding 6%. Three of the results had a crack width of less than 0.17 mm. The maximum absolute error among specimens with a crack width of 0.170 mm or less was observed to be within 0.014 mm. However, when calculating the relative error rate, a small reference value was applied to the denominator ($w_{m}$), resulting in a relatively high error rate. 

Overall, the estimation is deemed reasonable considering the relative and absolute error ranges. However, in the process of quantifying the geometry of the crack shape, the crack width and length were measured by 6-point arithmetic mean and visual observation, respectively. Since the crack depth was assumed to be the height of the specimen, errors may be involved.

The information about the crack in the specimen is limited to the parts observable with the naked eye; hence, capturing the effect of the internal shape of the specimen crack is challenging. If the actual crack geometry is considered in the calculation process, the estimated crack width could vary depending on the shape of the crack. 

To analyze the effect when accurate crack information is considered, we considered the following studies. In Section 4.2, the effect of lateral crack shape was examined. The crack area of the specimen was accurately calculated by image analysis, and the crack width was recalculated using the crack area. Section 4.3 investigates the effect of the internal vertical crack shape, which cannot be observed with the microscope. As shown in Figure 5, the crack length in the y-direction was defined as the crack length, and the vertical crack length in the z-direction was defined as the crack depth. 

### 4.2. Effect of Transverse Tortuosity of Crack

**Figure 9:**
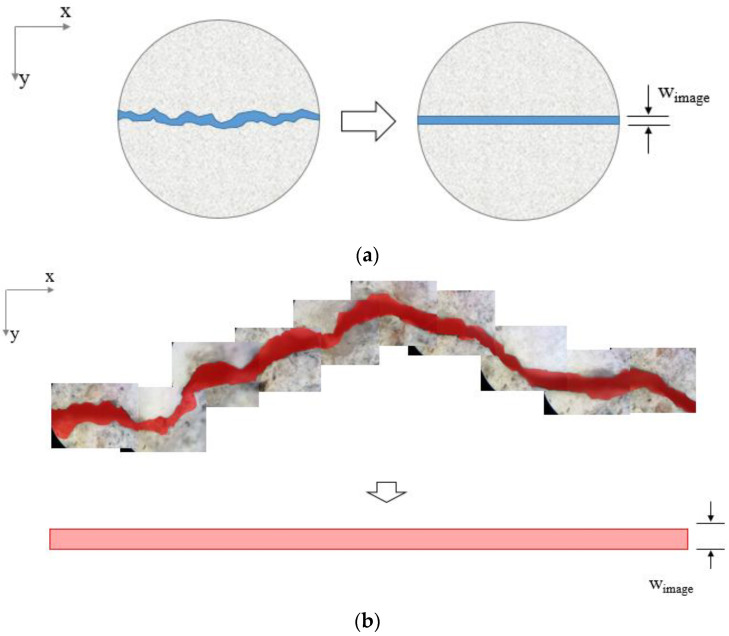
(**a**) Method for calculating average crack width based on image analysis and (**b**) optical photo of the specimen.

In a cracked specimen, as depicted in Figure 9, the area of the crack could vary depending on the shape of the crack. When the tortuosity of the crack increases, so does the crack’s path within the specimen, leading to an increase in the crack area. Inaccurate results would result from evaluating the diffusion coefficient or the crack width using only limited geometric information about the crack.

The actual area of the specimen’s crack could be determined by extracting image data from the crack. Accordingly, the precise average crack width could also be calculated, as shown in Figure 9. Using a digital optical microscope, we captured images of the crack surfaces of the four test specimens with the longest average crack length. As shown in Figure 9b, several photos with a size of 1024 μm × 768 μm were taken and combined into a single image to obtain a high-resolution image of the entire crack. The crack area was separated from the image based on image analysis techniques, and its area was calculated by measuring the number of pixels in the crack area. The crack width (*w_image_*) was recalculated by dividing the crack area by the straight crack length, as shown in Figure 9. The recalculated average crack width based on image analysis is shown in Table 3. 

The recalculated crack width did not differ significantly from that determined with a microscope (*w_m_*). The difference between these two crack widths is up to 16 μm. However, most crack widths calculated by image analysis were wider than those determined by a microscope. The fragments, pores, and tortuosity on the surface of the crack are believed to contribute to this effect.

When the recalculated crack width (*w_image_*) is compared to the estimated crack width (*w_p_*) from the diffusion test, the estimation errors increase from 4.56% to 4.83% on average than those with the measured crack width (*w_m_*). This indicates that the diffusion test’s error in estimating the crack’s width increased even when more precise crack information was considered. Particularly for the D_50_11 specimen, the error rate increased by more than 3.00%, from 1.53% to 4.81%.

However, an interesting trend was that all of the recalculated crack widths from the image analysis were larger than the estimates from the gas diffusion experiment, whereas the microscopically measured crack widths did not exhibit any trend with the estimations. This underestimation of the diffusion coefficient of the cracked specimen may result from the existance of other factors that influence the diffusion coefficien of the crackt. This error is presumed to have occurred as a result of overlooking the effect of tortuosity in the depth direction of the crack (longitudinal direction of the specimen), as discussed in Section 4.3.

### 4.3. Effect of Vertical Tortuosity of Crack

**Figure 10:**
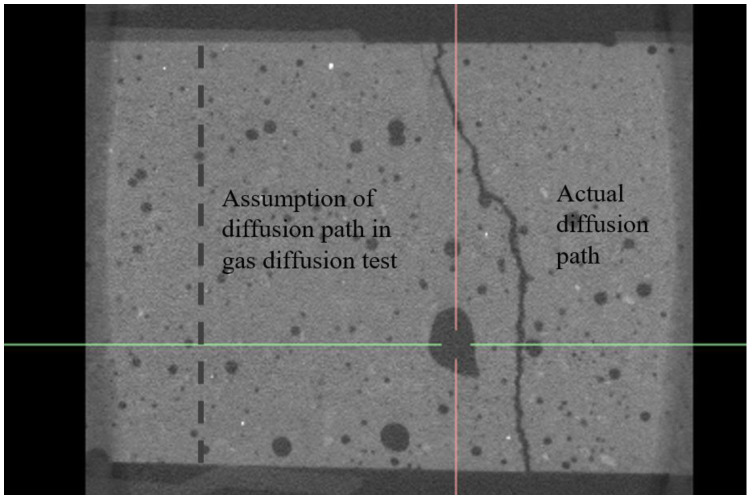
X-ray image of the internal crack shape of the specimen (x–z section).

The crack diffusion coefficient in Equation (1) was calculated assuming that the depth of the crack is equal to the thickness of the specimen from a practical point of view. However, the crack in the depth direction of the actual specimen may not always be as straight as assumed. Therefore, the effect on the crack diffusion coefficient was analyzed by measuring the crack characteristics along the specimen’s depth direction. The internal crack shape was measured using a 3D X-ray scanner, and the length of the crack path in the depth direction was determined by photographing the vertical cross-sections within the four selected specimens. Figure 10 shows a photograph of the internal crack observed in the x–z section of the D_50_11 specimen.

The actual crack path of the cracked specimen is longer than the thickness of the specimen due to the vertical tortuosity of the crack. Thus, due to the underestimation of the crack depth in Equation (1), the estimated crack widths from the diffusion experiment are typically less than the actual crack width (*w_image_*).

The internal crack shape was extracted from the x–z section image to determine the actual crack depth (vertical crack path length). Crack shapes were captured at multiple locations along the length of the crack. As shown in Table 4, the average crack depths were measured from these captured crosssections.

**Figure 11:**
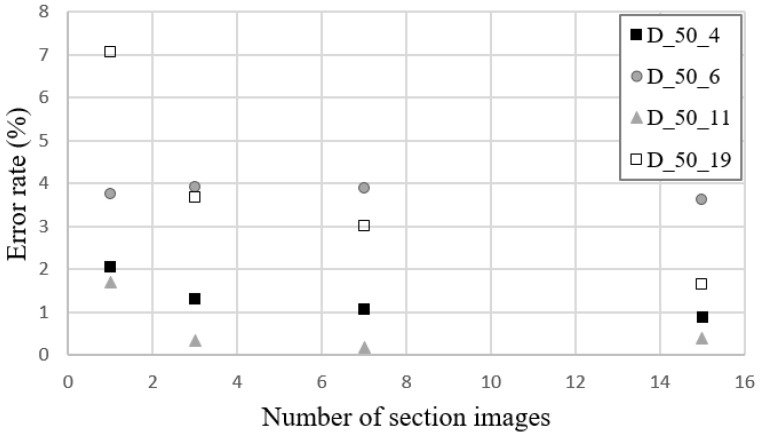
Error of the re-estimated crack width to the actual crack width as more images were applied for the calculation of average crack depth.

The crack diffusion coefficient and crack width were re-estimated by substituting the thickness of the specimen in Equation (1) with the crack depth calculated by X-ray analysis. Figure 11 depicts the deviation between the recalculated and actual crack widths (*w_image_*). As depicted in the figure, the error between the estimated crack width and the actual crack width tended to decrease as more cross-section images were used to calculate the average crack depth; that is, the crack depth was accurately measured.

Compared to the results presented in Section 4.1, the average estimation error decreased from 4.83% to 1.45%. These results suggest that gas diffusion phenomena are affected by the geometric shape of the crack, so the tortuosity of the crack in both the length and depth directions must be considered simultaneously to improve the estimation accuracy for a crack diffusion coefficient or a crack width. However, since the difference in error is not statistically significant (less than 3.38%), the specimen height may be substituted for the crack depth.

### 4.4. Application Examples 

Crack width is a significant durability indicator for concrete structures. The proposed test method could be used effectively to estimate the width of cracks in specimens made from a variety of materials. The method includes a procedure for compensating for the effects of diffusion occurring in the matrix other than cracks, so that it can be applied to a cement-based specimen.

The proposed test method is expected to be useful for determining the self-healing performance of cementitious materials by monitoring the variation of a crack’s width. As self-healing progresses, the cracks become narrower due to the filling of healing products. Typically, the healing performance of self-healing concrete can be evaluated using the reduction ratio of the crack diffusion coefficient, as demonstrated in [8,15,20].

## 5. Conclusions

This study proposed a gas diffusion test for estimating the crack width of a mortar specimen with cracks. Using the proposed method, we estimated the crack widths of mortar specimens, and the results were validated using the crack information obtained from an optical microscope and 3D CT scanner.

1. The test method is designed to induce diffusion phenomena by applying a concentration gradient to both sides of the specimen without a pressure gradient. It has been demonstrated that diffusion occurs both through the crack and the cement matrix. Because diffusion through the cement matrix influences the overall diffusion of a cracked specimen, a method is proposed for measuring the amount of oxygen diffusing solely through the crack area.
2. A pre-stabilization is incorporated into the testing procedure to reduce the duration of the diffusion experiment. By conducting a 24 h pre-stabilization, we can shorten the main diffusion experiment to approximately 1 h without sacrificing precision.
3. The crack diffusion coefficient is proven to be proportional to the crack width. Using the proposed method, this study demonstrated that the crack width of a 50 mm specimen could be estimated with a relative error of approximately 4%. The error in estimating the width of a crack was analyzed. Accurate consideration of the geometric characteristics of the crack reduced the estimation error. However, the 6-point average crack width and the specimen height could be used to reasonably represent a crack’s width and depth, as the difference in error is insignificant.
4. Given that the amount of permeation of harmful substances depends on the width of the crack, it is essential to measure the crack width rationally. Due to the low viscosity of the gas, the proposed gas diffusion experiment could be used effectively to estimate the crack width of a mortar specimen, even for a narrow crack width. Even so, it is anticipated that the proposed test method will be useful for monitoring the variation of a crack’s width to estimate the self-healing performance of cementitious materials.

## Figures and Tables

**Table 1:** Specimen information.

Series	Specimens ID	Crack Width (mm)	Crack Length (mm)	Thickness (mm)
D_25	D_25–01–09	0.150–0.650	60–65	25
D_50	D_50–01–20	0.150–0.450	70–75	50

**Table 2:** Comparison of the measured and estimated crack width.

Specification		Crack Width (μm)			
		Measured (*w_m_*)	*Estimated (w_p_)*	$\left|w_{m}-w_{p}\right|$	$\left|\frac{w_{m}-w_{p}}{w_{m}}\right|$
D_25	1	160	154	6	3.90%
	2	173	168	4	2.37%
	3	249	258	9	3.49%
	4	274	263	11	4.18%
	5	301	313	12	3.83%
	6	324	314	10	3.18%
	7	440	465	25	5.38%
	8	471	484	13	2.69%
	9	641	627	14	2.23%
D_50	1	152	138	14	9.21%
	2	141	131	10	7.09%
	3	162	154	8	4.94%
	4	119	127	8	6.72%
	5	124	120	4	3.23%
	6	231	238	7	3.03%
	7	274	277	3	1.09%
	8	274	264	10	3.65%
	9	252	257	5	1.98%
	10	261	257	4	1.53%
	11	277	258	19	6.86%
	12	313	309	4	1.28%
	13	308	317	9	2.92%
	14	191	207	16	8.38%
	15	276	260	16	5.80%
	16	425	444	19	4.47%
	17	340	335	5	1.47%
	18	431	424	7	1.62%
	19	415	402	13	3.13%
	20	398	390	8	2.01%

**Table 3:** Crack width recalculation considering the actual shape of the crack.

Specimens ID	Crack Length (mm)			Crack Width (μm)		
	Top	Bottom	Average	*w_m_*	*w_image_*	*w_p_*
D_50_4	73.4	71.5	72.45	119	135	127
D_50_10	72.9	72.2	72.55	261	270	257
D_50_11	71.8	74.0	72.90	277	269	258
D_50_19	73.7	73.0	73.35	415	421	402

**Table 4:** Average crack depth to the number of section images.

Specimens ID	Average Crack Depths (mm)			
	1 Section	3 Sections	7 Sections	15 Sections
D_50_4	54.25	52.45	52.58	52.67
D_50_10	51.54	51.50	51.51	51.58
D_50_11	52.58	52.04	52.18	52.03
D_50_19	53.55	52.98	52.87	52.64

## Data Availability

Data will be made available on request.
